# Family function fully mediates the relationship between social support and perinatal depression in rural Southwest China

**DOI:** 10.1186/s12888-021-03155-9

**Published:** 2021-03-12

**Authors:** Yilin Huang, Yan Liu, Yu Wang, Danping Liu

**Affiliations:** 1 West China School of Public Health and West China Fourth Hospital, Sichuan University, Chengdu, 610041 China; 2 Department of Radiation Oncology, National Cancer Center and Cancer Hospital, Peking Union Medical College, Chinese Academy of Medical Sciences, Beijing, China

**Keywords:** Social support, Family function, Perinatal depression, Rural China

## Abstract

**Background:**

Perinatal depression is the most common complication of gestation and childbearing affecting women and their families, and good social support and family function are considered protective and modifiable factors. This study aimed to investigate depression status and explore inter-relationships between social support and perinatal depression considering the influence of family function in rural areas of Southwest China.

**Methods:**

This is a cross-sectional study. The following instruments were used: the Edinburgh Postpartum Depression Scale, the APGAR Family Care Index Scale, and the Social Support Rate Scale. A structural equation modelling was used to test the hypothesis relationships among the variables.

**Results:**

A total of 490 rural antenatal (*N* = 249) and postpartum (*N* = 241) women (mean age (standard deviation), 28.17 ± 5.12) participated. We found that the prevalence of depression symptoms was 10.4%. Path analysis showed that family function had a direct negative correlation with depression (β = − 0.251, 95%CI: − 0.382 to − 0.118). Social support had a direct positive correlation with family function (β =0.293, 95%CI: 0.147 to 0.434) and had an indirect negative correlation with depression (β = − 0.074, 95%CI: − 0.139 to − 0.032), family function fully mediated the relationship between social support and depression.

**Conclusions:**

Findings of this study highlight that family function should be considered as the key target for interventions aiming to lower the prevalence of perinatal depression. Family members interventions are critical to reduce depression among antenatal and postpartum women.

**Supplementary Information:**

The online version contains supplementary material available at 10.1186/s12888-021-03155-9.

## Background

The perinatal period is an important time of family transition for women and is associated with an increase in the onset of new or recurrent mental disorders. Depression and anxiety are the most common mental disorders experienced during the perinatal period [1, 2]. Perinatal depression is of concern not only because of the suffering and distress it causes for women but also because of the risk of adverse effects on the developing foetus and child [3, 4].

The prevalence of perinatal depression in China has been estimated at 16.3% in a recent meta-analysis, with trends suggesting an increasing prevalence over the last decade and in less developed regions [5]. Perinatal depression usually has its onset during the third trimester of pregnancy or in the postpartum, affecting one in seven women [6, 7]. The prevalence of antenatal depression is 19.7% in China [5]; this can be partly explained by the negative effects of psychosocial changes during pregnancy as well as various hormonal factors [8–10]. The prevalence of postpartum depression is 14.8% in China and there is a rising trend thereof [5]. A study has shown that women were more vulnerable to psychiatric illness after birth [11].

Women with antenatal depression appear to be at considerably higher risk of several negative outcomes: self-harm or suicide; failure to seek prenatal care; and, poor diet [12, 13]. In turn, this may lead to adverse pregnancy outcomes such as complications during pregnancy, premature birth, dysplasia of the foetus and low infant birth weight [14, 15].. Additionally, postpartum depression is also associated with shorter breastfeeding duration [16] and may also be a risk factor for low social capacity in children [17]. Existing literature shows that factors associated with pregnancy and postpartum depression mainly include socio-demographic characteristics including maternal age, marital status, education, income and complications of pregnancy [15, 18–20] as well as social support [21], and family function [22].

Social support is defined as instrumental, informational, and emotional support provided by a social network including family, friends, and neighbourhoods, which can safeguard psychological well-being through buffering the effects of traumatic life events [23]. It can be characterised by the provider of support, including support from a spouse, relatives, or friends, with each thought to have independent protective effects against depression [24]. Social support as a protective and modifiable factor has been well investigated in relation to antenatal or postpartum depression [25]. Some studies have also shown that low-levels of social support were risk factors for perinatal depression [26, 27]. With adequate support, family ties will strengthen as has been shown in studies by Tarkka et al. (2003) and Lepistöet al. (2017) which regard social support as an important resource to improve family functioning [28, 29].

Family functioning can be defined as the degree to which a family performs as a unit to manage conditions, activities, external stimuli, or events that cause stress [30]. Compared to healthy families, families with family dysfunction are characterised by having lower cohesion, lower warmth, and lower expressiveness but also higher conflict, rigidity, and affectionless control [22]. Previous studies have shown that depression is negatively correlated with family functioning [31, 32]. A study undertaken in China demonstrated that stronger family support can help improve the mental health of pregnant women [33]. In addition, Wang et al. (2019) proposed a model that family function moderated the indirect relationship between social support and depression among the elderly [34].

**Fig. 1:**
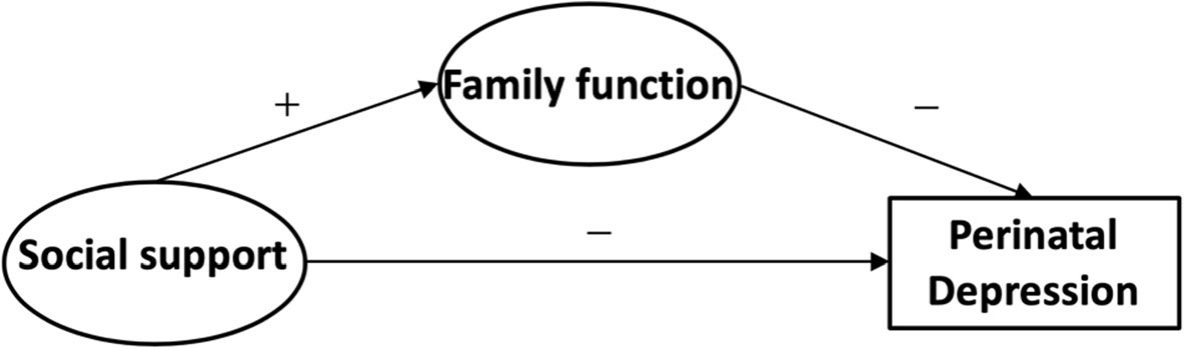
The theoretical model and hypothesis

The availability of mental health resources in rural areas of China is low [35]. Studies have shown that living in rural areas of China is very significantly associated with perinatal depression [36]. Despite previous studies which have demonstrated the relationship between family function and depression as well as social support and depression, few studies have included these three variables in one study. We do so here in order to understand the interrelationships and potential mechanisms of social support, family function, and perinatal depression. We examined the influence of social support and family function on perinatal depression in rural areas of southwest China in this study. Based on the above description, we hypothesise a single mediator model as shown in Fig. 1. Specifically, social support would be positively associated with family function (Hypothesis 1) and negatively associated with depression (Hypothesis 2). We also hypothesise that family function would be negatively associated with depression (Hypothesis 3). Furthermore, we suggested that the relationship between social support and depression would be mediated by the family function (Hypothesis 4). The study aims to assess the prevalence of perinatal depression in rural China and to identify key factors including social support and family function which contribute to the prevention and control of perinatal depression.

## Methods

### Participants and procedure

This cross-sectional study was conducted among pregnant and postpartum women in rural areas of Sichuan Province, Southwest China, from December 2017 to May 2018. The optimal time to conduct the first screen for postpartum depression is within 6 months postpartum [37, 38]. Therefore the target population in this study was the women who were pregnant or within 6 months postpartum.

A multi-stage stratified random sampling was used to acquire the sample. In the first stage, we randomly chose a city in Sichuan province. In the second stage, we randomly selected a rural district in the city. In the third stage, 10 townships were randomly selected from the rural district. In the fourth stage, we randomly selected 50 maternal women from the database of maternal women established by each township hospital. Trained investigators invited the selected participants to take part in a face-to-face interview in their home and the questionnaires were completed by the investigators. We used the quantifiable scales, trained investigators, two-person data entry, and logical verification to ensure the quality of the research.

### Ethical considerations

The study protocol was approved by the Institutional Review Board of Sichuan University (Project identification code: H171260). The study was explained by the trained investigators to participants and informed written consent was obtained within 10 min of consideration before data collection.

### Measures

Participants’ socio-demographic characteristics, social support, family function, and depression information were collected from questionnaires.

#### Socio-demographic characteristics

Socio-demographic characteristics included age, perinatal status, marital status, education level, employment status, individual annual income, medical insurance status, and complications of pregnancy.

#### Social support

Social support was assessed through the Social Support Rating Scale (SSRS), which was developed by Xiao (1994) [39]. The SSRS was specifically designed for use in a Chinese context and consists of ten items of three domains in total: objective support, subjective support, and social support utilization. Responses were provided as a 4-point Likert scale. The overall score of all items ranges from 12 to 66 with higher scores reflecting stronger social support. The total score has been divided into three levels: low (12–22), moderate (23–44), and high (45–66). The SSRS has been widely applied in China with excellent validity and reliability [40, 41]. In this research, Cronbach’s α of the scale was 0.825.

#### Family function

Family function was measured by the APGAR, developed by Smilkstein [42], which was used to evaluate an individual’s satisfaction with family function. This scale is a 3-point scale ranging from 0 (hardly ever) to 2 (almost always), composed of five items: adaptation, partnership, growth, affection and resolve. The total score ranges from 0 to 10 with higher scores denoting a higher level of satisfaction with family function. It is generally believed that scores of 0–3 indicate severe family dysfunction, scores of 4–6 indicate moderate family dysfunction, and scores of 7–10 indicate good family function. The Chinese version of APGAR has been widely applied in China with excellent validity and reliability [43, 44]. In this research, Cronbach’s α of the scale was 0.874.

#### Depression

Depression was measured by the Edinburgh Postnatal Depression Scale (EPDS). The EPDS, designed by Cox, et al. (1987) [45], was originally developed to assist primary care health professionals to detect mothers suffering from postpartum depression and was also proved to be suitable for the detection of antenatal depression in 2003 [46]. The EPDS is a 10-item self-reported questionnaire on depressive symptoms. Each item is scored on a 4-point scale (from 0 to 3), so that the total score ranges from 0 to 30, with higher scores representing more depressive symptoms. The EPDS was translated into a Chinese version by Pen et al. in 1994 [47], who recommended that the cut-off score for the Chinese was 9.5, and the score of 9.5 or higher indicates significant depressive symptoms. In this research, Cronbach’s α of the scale was 0.776.

### Statistical analyses

The data were entered using the Epidata3.1 database and were analysed using SPSS version 20.0 (SPSS Inc., Chicago, IL, USA) and Analysis of Moment Structures (AMOS) version 24.0 (IBM, New York, NY, USA). First, we calculated descriptive statistics (frequencies, percentages, means, and standard deviations) to examine the socio-demographic characteristics of the sample. Second, we undertook a descriptive analysis of study variables (means and standard deviations). Third, binary logistic regression models were used to test the relationship between social support, family function, and depression. In model 1, we used depression as the dependent variable and social support, socio-demographic variables as independent variables. In model 2, we further added the family function as an independent variable. Fourth, a structural equation model (SEM) was employed to further test the hypothesis relationships among social support, family function, and perinatal depression.

The SEM used bootstrap maximum likelihood estimation and results with a *p*-value of < 0.05 were considered statistically significant. To examine the model fit, we employed several indicators with their cut-offs: adjusted goodness of fit index (AGFI), a goodness of fit index (GFI), the comparative fit index (CFI), normed fit index (NFI), incremental (IFI), and Tucker-Lewis index (TLI) of 0.90 or above; a root mean squared error of approximation (RMSEA) less than or equal to 0.08, indicated an acceptable model fit [48].

## Results

### Participants and socio-demographic characteristics

Of the 500 participants invited to take part, 498 agreed and returned questionnaires with a response rate of 99.6%. Questionnaires were checked after the interviews for completeness. Eight records met exclusion criteria (Incomplete data collection: *n* = 2; postpartum period> 6 months *n* = 6). Overall, 490 questionnaires were valid.

Socio-demographic characteristics of the 490 samples are shown in Table 1. The proportion of antenatal women and postpartum women were 50.8 and 49.2%, respectively. The mean age (standard deviation) was 28.17 ± 5.12, ranging from 19 to 43 years. Most were married (96.7%), educated at high school or vocational school level or less (73.7%). The majority of the women were currently unemployed (57.3%), had an individual annual income of less than $750 (41.2%), and held medical insurance (98.0%). Most had no complications of pregnancy (81.2%).

**Table 1:** Socio-demographic characteristics of the sample (*n* = 490)

Socio-Demographic Characteristics	***N (%)***
Age, mean ± SD	28.17 ± 5.12
perinatal status	
Antenatal women	249 (50.8%)
Postpartum women	241 (49.2%)
Marital status	
Married	474 (96.7%)
Unmarried/ Divorced/ Widowed	16 (3.3%)
Education level	
Elementary and below	31 (6.3%)
Middle school	141 (28.8%)
High or vocational school	189 (38.6%)
College and above	129 (26.3%)
Employment status	
Employment	209 (42.7%)
Unemployed	281 (57.3%)
Individual annual income, ($)	
<750	202 (41.2%)
750 ~ 1499	68 (13.9%)
1500 ~ 4499	122 (24.9%)
4500 ~ 7499	73 (14.9%)
≥ 7500	25 (5.1%)
Medical insurance	
No	10 (2.0%)
Yes	480 (98.0%)
Complications of pregnancy	
No	398 (81.2%)
Yes	92 (18.8%)

### Descriptive analysis of study variables

Table 2 shows scores of social support, family function, and depression. The mean score (standard deviation) of social support was 40.79 ± 5.95 and 0.2% (1), 71.6% (351), and 28.2% (138) of participants had low, moderate, and high social support, respectively. The mean score (standard deviation) of family function was 8.80 ± 1.89; 85.5% (419) of participants had good family function while 13.1% (64) and 1.4% (7) of participants experienced moderate and severe family dysfunction, respectively. The mean score (standard deviation) of depression was 5.30 ± 3.46, and 10.4% (51) of women had significant depression symptoms. The mean score (standard deviation) of depression among antenatal and postpartum women were 5.78 ± 3.30 and 4.80 ± 3.57, respectively; 10.4% (26) of antenatal women and 10.4% (25) of postpartum women had significant depression symptoms. ANOVA showed that social support and family function were significantly correlated with depression symptoms.

**Table 2:** Description of social support, family function scores with and without depression symptoms (*n* = 490)

Contents	Range	Total Mean (SD)	Depression (EPDS< 9.5, *n* = 439) Mean (SD)	Non-depression (EPDS≥9.5, *n* = 51) Mean (SD)	*p*-Value
Social support	12—66	40.79 (5.95)	38.76 (5.40)	41.03 (5.97)	0.009**
Objective support	1—22	9.8 (2.21)	9.06 (2.10)	9.89 (2.20)	
Subjective support	8—32	22.89 (3.98)	22.29 (4.25)	22.96 (3.94)	
Support utilization	3—12	8.10 (1.70)	7.41 (1.64)	8.18 (1.69)	
Family function	0—10	8.80 (1.89)	7.37 (2.50)	8.97 (1.74)	< 0.001**
Adaptation	0—2	1.77 (0.45)	1.43 (0.57)	1.81 (0.42)	
Partnership	0—2	1.74 (0.48)	1.43 (0.57)	1.77 (0.46)	
Growth	0—2	1.74 (0.48)	1.43 (0.64)	1.77 (0.44)	
Affection	0—2	1.73 (0.49)	1.51 (0.54)	1.76 (0.48)	
Resolve	0—2	1.82 (0.41)	1.57 (0.61)	1.85 (0.37)	
Depression	0—30	5.30 (3.46)	11.76 (3.18)	4.55 (2.61)	
Antenatal	0—30	5.78 (3.30)	12.38 (3.89)	5.00 (2.18)	
Postpartum	0—30	4.80 (3.57)	11.12 (2.11)	4.07 (2.92)	

Notes: ** *p* < 0.05

### Binary logistic regression analyses of depression

Table 3 shows the results of the binary logistic regression analyses testing the relationship between social support, family function, and depression. The results of model 1 suggest that there was no other factor except for social support which was significantly correlated with depression (AOR = 0.933, *p* = 0.012). The results of model 2 suggest that only family function was significantly correlated with depression (AOR = 0.720, *p* < 0.001). According to the collinearity diagnosis, the VIF of each regression coefficient was no more than 2, the model fitted well. After controlling for the influence of family function, a previously significant relationship between social support and depression became non-significant, suggesting a full or perfect mediation relationship existed.

**Table 3:** Binary logistic regression of factors associated with the depression

Factors	Model 1			Model 2		
	AOR	***p***-Value	95%CI for AOR	AOR	***p***-Value	95%CI for AOR
Social support	0.933	0.012**	(0.884,0.985)	0.945	0.060	(0.892,1.002)
Family function				0.720	< 0.001**	(0.628,0.824)
Age	0.985	0.669	(0.919,1.056)	0.992	0.830	(0.924,1.066)
Perinatal status (ref: Antenatal women)						
Postpartum women	0.902	0.744	(0.484,1.680)	1.130	0.713	(0.589,2.171)
Marital status (ref: Married)						
Unmarried/Divorced/Widowed	0.607	0.573	(0.069,4.785)	0.617	0.659	(0.072,5.257)
Education level (ref: Elementary and below)						
Middle school	2.435	0.415	(0.287,20.662)	2.797	0.357	(0.314,24.940)
High or vocational school	4.017	0.199	(0.482,33.489)	4.790	0.158	(0.544,42.186)
College and above	3.837	0.220	(0.447,32.909)	4.871	0.159	(0.537,44.192)
Employment status (ref: Employment)						
Unemployed	1.200	0.580	(0.629,2.292)	1.343	0.388	(0.688,2.624)
Individual annual income (ref: <750, $)						
750 ~ 1499	0.690	0.483	(0.244,1.947)	0.720	0.548	(0.247,2.099)
1500 ~ 4499	1.320	0.458	(0.634,2.745)	1.591	0.232	(0.743,3.406)
4500 ~ 7499	0.967	0.944	(0.373,2.505)	0.997	0.995	(0.373,2.661)
≥ 7500	0.324	0.298	(0.039,2.708)	0.342	0.361	(0.034,3.418)
Medical insurance (ref: NO)						
Yes	144,344,517.3	0.999	(0.000)	98,701,368.48	0.999	0.000
Complications of pregnancy (ref: No)						
Yes	1.484	0.273	(0.733,3.007)	1.300	0.482	(0.626,2.701)

Notes: *AOR* means adjusted odds ratio, ** *p* < 0.05

### Test of study model

**Fig. 2:**
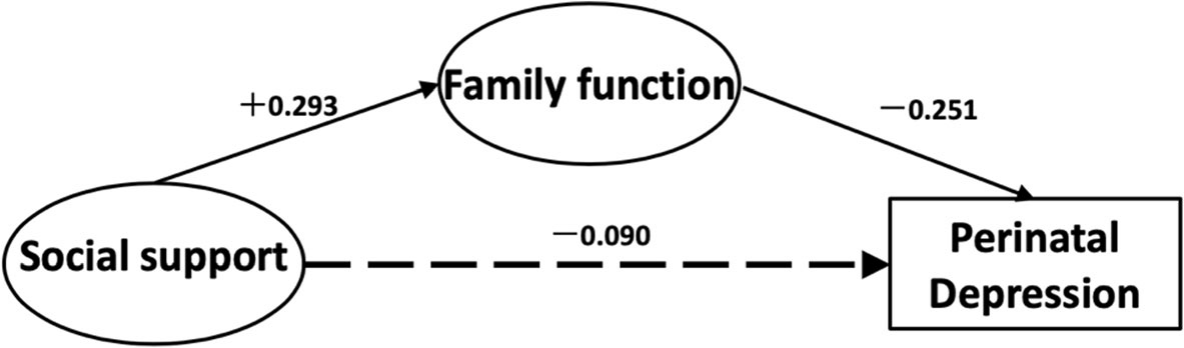
The final model and standardized model paths

Figure 2 shows path analysis testing results of the fitness of the hypothetical model in Fig. 1. The final model had an adequate fit: GFI = 0.960, AGFI = 0.928, NFI = 0.934, IFI = 0.951, TLI = 0.928, RMSEA = 0.075.

The estimates for direct, indirect, and total effects with bias-corrected 95% CI are shown in Table 4. In these analyses, effect coefficients are substantially significant if the 95% CI does not include 0. The results showed that social support had a significant positive correlation with family function (β =0.293, 95%CI: 0.147 to 0.434), thus supporting Hypothesis 1. However, the direct impact of social support on depression proved to be statistically non-significant (β = − 0.090, 95%CI: − 0.213 to 0.043), leading us to reject Hypothesis 2. The family function had a direct negative correlation with depression (β = − 0.251, 95%CI: − 0.382 to − 0.118), thus supporting Hypothesis 3. In addition, social support had an indirect negative correlation with depression (β = − 0.074, 95%CI: − 0.139 to − 0.032), thus supporting Hypothesis 4.

**Table 4:** Direct, indirect and total effects and 95% confidence intervals for the final model

Model pathways	Estimated effect	95%CI
Total effects		
Depression <−-- Social support	−0.164	−0.274 to 0.028
Family function <−-- Social support	0.293	0.147 to 0.434
Depression <−-- Family function	−0.251	−0.382 to −0.118
Direct effects		
Depression <−-- Social support	−0.090	−0.213 to 0.043
Family function <−-- Social support	0.293	0.147 to 0.434
Depression <−-- Family function	−0.251	−0.382 to − 0.118
Indirect effects		
Depression <−-- Social support	−0.074	− 0.139 to − 0.032

Regarding the path between social support and depression, the total effect and indirect effect were statistically significant but the direct effect was statistically non-significant. Based on the above, family function fully mediates the relationship between social support and depression.

## Discussion

This study aimed to investigate depression status and clarify the inter-relationships between social support and depression considering the influence of family function among perinatal women in rural areas of Southwest China. To our knowledge, this is the first study to report the fully mediating role of family function between social support and perinatal depression. The findings of this study give important implications of development and implementation of interventions to ameliorate perinatal depression for mothers’ health and to promote the future wellbeing of their children and families in rural areas.

The women in this study were investigated in communities and the prevalence of perinatal depression symptoms was 10.4%, which is close to a previous meta-analysis which found a pooled prevalence of 10.7% in community settings [5]. In this meta study, the pooled prevalence in hospital settings was 17.6%, which is higher than the prevalence in our study [5]. The reason may be that most studies in hospital settings reported women within 6 weeks postpartum. Women during the 6 weeks postpartum are required to engage in certain practices to promote the health of the maternal/new-born dyad for the Chinese tradition of “doing the month”, mainly including promoting maternal rest, reducing domestic duties, and restricting activities at home [49]. To coerce a person into a certain behaviour, even if it is apparently for their good, is classified as negative and ineffective support [50]. Due to the limitation in physical and social activities, with frustrations around breastfeeding and lack of sleep which cannot be relieved effectively, women may be stressed which might lead to mood alterations [51]. After the first 6 weeks postpartum, with social activities resumed, women may accommodate and gradually accept their new situation [52].

The mean family function (APGAR) score was 8.80 ± 1.89, and only 1.4% of women reported severe family dysfunction in our study. A possible reason may be that in traditional rural China, pregnancy is regarded as a great familial contribution, and family members will acknowledge the women’s family status and strive to develop better family function [53]. The model revealed that perinatal women with lower family function were more likely to experience depression symptoms, which is consistent with previous studies [32, 54]. There are two possible explanations. One is that the couple relationship which plays an important role in family function will be affected in the perinatal period by more emerging work-family and economic conflicts [55] and fewer opportunities for shared intimacy, which leads to women experiencing lower moods [56]. Another possible explanation is that women in dysfunctional families communicate their emotions and thoughts ineffectively with other family members, thus leading to the development of depression [57].

The results revealed that the mean score of social support among antenatal and postpartum women was 40.79 ± 5.95, which is lower than another study (43.34 ± 7.06) in China that surveyed women before pregnancy [58]. A possible reason may be that the women after pregnancy decrease physical exercise and leisure activities due to the concerns of maternal/child health, and thus receive less social support [59]. Our model revealed that better social support predicted better family function which is consistent with Jiang et al. (2015) study [60]. There are two possible reasons. One reason may be that social support improves an individual’s physical health (for example, by increasing healthy activities and protective behaviours and promoting a healthier lifestyle) and ability to manage their stress and cope effectively; accordingly, the individual functions better within the family [61]. The other reason may be that the family members provide the most solid support in one’s social network [62], partly owing to Chinese culture-specific norms of reciprocal filial piety [63], therefore, good social support means good family function in Chinese societies.

The most significant finding of this study was that the relationship between social support and depression was fully mediated by family function. Previous studies identified that social support had a direct effect on depression [26, 64], but this research further found the effect was indirect. Our model reveals that the higher social support among perinatal women was less likely to experience depression symptoms which is consistent with previous studies, but interestingly, the association was fully mediated by family function. The family function is the key factor. This can be explained by the vulnerability-stress model, when perinatal women facing the stressor, the low social support leads to family dysfunction which increases environmental vulnerability and triggers the onset of depression [65]. Compared to western women, Chinese women seem to be more family-oriented and thus are more likely to be affected by family relationships [33].

The findings of this study highlight the importance of family function in decreasing perinatal depression in rural areas and have important implications for public health practices. Healthcare professionals should pay more attention to evaluating family function constantly across the perinatal period and take a partner-inclusive intervention to lower the risk of perinatal depression [66]. Combining assessments like APGAR, especially applying the simple Resolve item—“Are you satisfied with the way you and your family share time together?”- can help professionals quickly assess family function [67]. For the dysfunctional family, health professionals should focus on interventions including family members in addition to perinatal women, such as requiring family members to participate in prenatal health and baby care education, providing different types of health education programs for different family members and setting up consulting platforms of perinatal nursing for families [68]. These are beneficial for minimizing the harmful effects of family dysfunction.

Limitations of this study need to be recognized. Firstly, we cannot make claims about causality among the three variables because of the cross-sectional design. Future longitudinal or experimental studies should be conducted to provide causal inference. Secondly, some factors such as life stress, personal history of depression, and family history of depression have not been taken into consideration, which may also influence the depression level of perinatal women. In addition, the EPDS is a screening tool rather than a diagnostic tool, which can only provide information on symptoms of depression. Finally, although our study concerned people in the community, which could reduce selection bias, the data was obtained in rural areas of southwest China, so we should be careful when generalizing findings.

## Conclusions

This study investigates the interplay between social support and perinatal depression considering the influence of family function. Results suggest that family function plays a fully mediating role in the association between social support and depression. Findings of this study highlight that family function should be considered as the key target for interventions aiming to lower the prevalence of perinatal depression. Family members’ interventions are critical to reducing perinatal depression.

## Supplementary Information

**Additional file 1.** Perinatal Questionnaires.

**Additional file 2.** dataset(CSV 32KB).

## Data Availability

The datasets used and/or analysed during the current study available from the corresponding author on reasonable request.

## Abbreviations

SEM: Structural equation modeling
EPDS: Edinburgh Postnatal Depression Scale
APGAR: APGAR Family Care Index Scale
SSRS: Social Support Rate Scale
