# Supplementary material for: Family function fully mediates the relationship between social support and perinatal depression in rural Southwest China
Source: BMC Psychiatry. 2021 Mar 12;21:151. doi: 10.1186/s12888-021-03155-9 (PMC7953569; doi:10.1186/s12888-021-03155-9)
Supplement: Supplementary file 1 — Additional file 1. Perinatal Questionnaires. [file 12888_2021_3155_MOESM1_ESM.pdf]

Questionnaires

Part 1: Questions of socio-demographics

1.Your type of maternal women：

1）Antenatal women   2）Postnatal women (postpartum____months)

2.Your age is _____：

3.Your marital status：

1）Married   2）Unmarried/ Divorced/ Widowed

4.Your education level:

1）Elementary and below   2）Middle school

3）High or vocational school   4）College and above

5.Your employment status:

1）Employment   2）Unemployed

6.Your Individual annual income in 2017 (¥):

1）<5,000   2）5,000-9,999   3）10,000-29,999   4）30,000-49,999   5）≥50,000

7. Do you have any kind of medical insurance?

1）No   2）Yes

8. Do you have any complication of pregnancy? (e.g., pregnancy hypertension, gestational diabetes, anemia, heart disease during pregnancy, hyperthyroidism during pregnancy, pregnancy with hepatitis)

1）No   2）Yes

Part 2: Questions on social support (SSRS)

1. How many close friends whom you can get support and help from do you have?

1） None   2）1~2   3）3~5   4）6 or above 6

2. Over the past year, you:

1） lived alone and were away from families

2）often changed the residence and lived with strangers for most of the time

3）lived with classmates, colleagues or friends

4）lived with families

3. You and your neighbors:

1） never care about each other

2） may care slightly when meet with difficulties

3） some neighbors very care about you

4） most of neighbors very care about you

4. You and your classmates/colleagues:

1） never care about each other

2） may care slightly when meet with difficulties

3） some classmates/colleagues very care about you

4） most of classmates/colleagues very care about you

5.1 You get support and care from your spouse (lover).

1） None   2）Very little   3）General   4）Fully support

5.2 You get support and care from your parents.

1）None   2）Very little   3）General   4）Fully support

5.3 You get support and care from your children.

1）None 2）Very little 3）General 4）Fully support

5.4 You get support and care from your siblings.

1）None 2）Very little 3）General 4）Fully support

5.5 You get support and care from other family members (such as sisters-in-law).

1）None 2）Very little 3）General 4）Fully support

6. In the past, when you were in a difficult situation, the source of the economic support and practical help was from:

1）None

2）The following (can select multiple items):

A. Spouse B. Other family members C. Friends D. Relatives

E. Classmates/Colleagues F. Company/School G. Official or semi-official organizations

H. Unofficial organizations I. Others______

7. In the past, when you were in a difficult situation, the source of comfort and concern was from:

1）None

2）The following (can select multiple items):

A. Spouse B. Other family members C. Friends D. Relatives

E. Classmates/Colleagues F. Company/School G. Official or semi-official organizations

H. Unofficial organizations I. Others______

8. When you are in a trouble, you talk to:

1）Nobody 2）1~2 people who is/are very close to you

3）friends if they ask you 4）people initiatively

9. When you are in trouble, you ask for help:

1）Never 2）Occasionally 3）sometimes 4）Frequently

10. You join group organizing activities (such as party organizations, religious organizations, trade unions, student unions, etc.).

1)Never 2)Occasionally 3)Frequently 4)Initiatively and actively

Part 3: Questions on Family function (APGAR)

1 Almost always 2 Some of the time 3 Hardly ever

Please tick the answer that best suits your situation based on your family situation:	1	2	3
1.I am satisfied with the help that I receive from my family when something is troubling me.			
2. I am satisfied with the way my family discusses items of common interest and shares problem solving with me.			
3.I find that my family accepts my wishes to take on new activities or make changes in my life-style.			
4.I am satisfied with the way my family expresses affection and responds to my feelings such as anger, sorrow, and love.			
5. I am satisfied with the amount of time my family and I spend together.			

Part 4: Questions on depression (EPDS)

1 Never 2 Rarely 3 Sometimes 4 Often

Please tick the answer that best suits your situation based on your situation in the past week:	1	2	3	4
1. I have been able to laugh and see the funny side of things.				
2. I have looked forward with enjoyment to things.				
3. I have blamed myself unnecessarily when things went wrong.				
4. I have been anxious or worried for no good reason.				
5. I have felt scared or panicky for not very good reason.				
6. Things have been getting on top of me.				
7. I have been so unhappy that I have had difficulty sleeping.				
8. I have felt sad or miserable.				
9. I have been so unhappy that I have been crying.				
10. The thought of harming myself has occurred to me.				
